# TOOLBOX: Strep-Tagged nano-assemblies of antibody-drug-conjugates (ADC) for modular and conditional cancer drugging

**DOI:** 10.3892/or.2021.8110

**Published:** 2021-06-09

**Authors:** Elisa Tremante, Leonardo Sibilio, Fabio Centola, Nadine Knutti, Gerd Holzapfel, Isabella Manni, Matteo Allegretti, Paolo Lombardi, Giuseppe Salvo, Loredana Cecchetelli, Karlheinz Friedrich, Joachim Bertram, Patrizio Giacomini

Oncol Rep 45: 77, 2021; DOI: 10.3892/or.2021.8028

Following the publication of the above article, the authors have requested a change in the authorship on the paper, and the revised list of authors is presented above; essentially, the ninth intended author, Giuseppe Salvo (G.S.), was inadverently omitted from the author list. G.S. contributed towards the T design and the preparation of the tagged ScFv. Therefore, the revised authors’ names and affiliations, as they should have been presented in the original version of this paper, are as follows:

ELISA TREMANTE^1^, LEONARDO SIBILIO^2,7^, FABIO CENTOLA^2,8^, NADINE KNUTTI^3,9^, GERD HOLZAPFEL^4^, ISABELLA MANNI^5^, MATTEO ALLEGRETTI^1^, PAOLO LOMBARDI^6^, **GIUSEPPE SALVO^2,10^**, LOREDANA CECCHETELLI^2^, KARLHEINZ FRIEDRICH^3^, JOACHIM BERTRAM^4^ and PATRIZIO GIACOMINI^1^

^1^Oncogenomics and Epigenetics, IRCCS Regina Elena National Cancer Institute, Rome; ^2^Ibi Lorenzini, Aprilia, Italy; ^3^University Hospital Jena, Institute of Biochemistry II, Jena; ^4^IBA GmbH, Göttingen, Germany; ^5^SAFU, IRCCS Regina Elena National Cancer Institute, Rome; ^6^NaxosPharma, Novate Milanese, Milan, Italy

*Correspondence to:* Dr Patrizio Giacomini. Oncogenomics and Epigenetics, IRCCS Regina Elena National Cancer Institute, Via Elio Chianesi 53, 00144 Rome, Italy, E-mail: patrizio.giacomini@ifo.gov.it

*Present address*: ^7^Menarini Biotech, Pomezia, Rome, Italy

*Present address*: ^8^Merck Serono Spa, Global Analytical Department, Guidonia Montecelio, Rome, Italy

*Present address*: ^9^University Hospital Jena Institute for Clinical Chemistry and Laboratory Diagnostics, Jena, Germany

*Present address*: ^10^External Quality Assurance (ExM), MSD Italia S.r.l., Via Vitorchiano 151, 00189 Rome, Italy

Furthermore, the Authors contributions section should be amended to read as follows:

**Authors' contributions**

ET and MA tested the TOOLBOX concept and performed the flow cytometry experiments. **LS, FC, GS and LC designed and prepared the tagged ScFv.** NK and KF designed and prepared the GFP promoter-reporter construct. GH and JB designed and manufactured Strep-Tactins. ET and IM performed animal studies. PL designed and manufactured NAX and NAXT compounds. PG conceptualized TOOLBOX and wrote the manuscript with the contribution of all authors. All authors have approved the final version of the manuscript.

All the authors agree with the inclusion of Giuseppe Salvo as an author on this paper, and are grateful to the Editor for allowing them the opportunity to publish this Corrigendum. Furthermore, they apologize to the readership of the Journal for any inconvenience caused.

